# Knowledge, Attitudes, and Practices Toward COVID-19 Among Construction Industry Practitioners in China

**DOI:** 10.3389/fpubh.2020.599769

**Published:** 2021-01-08

**Authors:** Linzi Zheng, Ke Chen, Ling Ma

**Affiliations:** ^1^College of Public Administration, Huazhong University of Science and Technology, Wuhan, China; ^2^Department of Construction Management, School of Civil and Hydraulic Engineering, Huazhong University of Science and Technology, Wuhan, China; ^3^The Bartlett School of Construction and Project Management, University College London, London, United Kingdom

**Keywords:** COVID-19, knowledge, attitude, practice, construction industry practitioner

## Abstract

The COVID-19 pandemic has put labor-intensive industries at risk, among which the construction industry is a typical one. Practitioners in the construction industry are facing high probabilities of COVID-19 transmission, while their knowledge, attitudes, and practices (KAP) are critical to the prevention of virus spread. This study seeks to investigate the KAP of construction industry practitioners in China through an online questionnaire survey conducted from 15 to 30 June 2020. A total of 702 effective responses were received and analyzed. The results revealed that: (1) although an overwhelming percentage of respondents had the correct knowledge about COVID-19, there were significant respondents (15% of all) who were unsure or wrong about the human-to-human transmission of the virus; (2) practitioners generally showed an optimistic attitude about winning the battle against the COVID-19 pandemic and were satisfied with the governments' contingency measures; (3) practitioners tended to actively take preventive measures, although checking body temperature, wearing face masks, and keeping safe social distance still needs to be reinforced. This research is among the first to identify the KAP of construction industry practitioners toward the COVID-19 pandemic in China. Results presented here have implications for enhancing strategies to reduce and prevent COVID-19 spread in the construction industry.

## Introduction

Starting from December 2019, a novel coronavirus disease (COVID-19) emerged in the global community. Up to 17 August 2020, there have been a total of 21,598,893 confirmed cases with 773,934 deaths (1). The pandemic has also seriously affected many industries, and the construction industry is no exception. Many construction projects in Wuhan, the epicenter of the COVID-19 outbreak in China, have been suspended because of the city-wide shutdown of all non-essential work and restriction of public transportation that led to a shortage of essential materials and skilled practitioners (2). Although regions are able to continue their construction projects, the inherent labor-intensive nature of construction project causes additional challenges due to the onsite necessity of construction task delivery and the constraints on the feasibility of social distancing on an active jobsite.

When dealing with the COVID-19 pandemic, the health and safety of employees supersedes other priorities. Different countries have developed a series of guidelines and suggestions for infection prevention. For instance, the Center for Disease Control and Prevention (CDCP) of the U.S. suggested that temporary, mobile handwashing stations should be installed if hand sanitizer and running water is not available on the construction site (3). The Chinese government suggested that frequently touched surfaces, such as shared tools and other equipment, should be cleaned and disinfected (4). Following these guidelines and suggestions, individuals might need to change their behaviors in daily operations. For example, they should wear face masks and keep social distancing in the workplace in order to interrupt the human-to-human transmission chain.

Knowledge, attitudes, and practices (KAP) theory suggested that the changes in human behavior can be divided into three successive processes: knowledge acquisition, attitude generation, and behavior formation (5). Based on this theory, people's adherence to COVID-19 control measures could be affected by their knowledge and attitudes. Several recent studies have reported the KAP of residents toward COVID-19 during the rapid rise period of the virus outbreak in China, Malaysia, the Philippines, etc. (6–9). Intensive research efforts have also identified the KAP status of medical professionals who work on the frontline to prevent the spread of COVID-19 (10–13). However, less attention has been paid to the construction industry where the practitioners are at considerable risk for severe illness from COVID-19. Therefore, at this critical moment, there is an urgent need to assess the KAP toward COVID-19 among construction industry practitioners. Such an investigation will not only identify knowledge gaps that could enhance the understanding for COVID-19 control efforts, but also set priorities to address the most common problems in protecting practitioners from being infected with the COVID-19 virus.

This study conducted a KAP survey to investigate the KAP toward COVID-19 among construction industry practitioners in China. Three specific research objectives will be attained:

1. To assess the knowledge of construction industry practitioners regarding the epidemiological features of COVID-19 and the prevention of infection;
2. To evaluate the attitudes of construction industry practitioners toward the control of the COVID-19 pandemic;
3. To identify the practices taken by construction industry practitioners regarding infection prevention.

In the next section, the protocol and process of the KAP survey are described. Section **Results** reports the main findings of the KAP assessment, and discussions of the findings are presented in Section **Discussion**. The last section concludes this study.

## Research Methods

### Survey Platform and Sampling

In this study, the KAP survey was conducted following the recommendations of the World Health Organization and existing studies on individuals' KAP toward COVID-19. The survey was conducted between 15 and 30 June 2020, and the questionnaire was distributed through the Tencent platform (https://wj.qq.com/). Considering the target population of this survey focused on practitioners in the construction industry, this study did not adopt a convenience sampling strategy, but the authors approached the survey participants based on their personal networks. Through the Tencent survey platform, the participants were first provided with a brief introduction of the survey, including the survey objectives, procedures, voluntary nature of participation, and declarations of confidentiality, before they decided whether or not to take this survey.

### Questionnaire

The main body of the questionnaire contained two sections. The first section collected socio-demographic information including gender, age, years of work experience, stakeholder, and the type and location of their engaged projects. Gender, age, years of work experience, and stakeholder were recorded as reported by the respondent. The type of project was classified as residential, commercial, industrial, infrastructure, and others; the location was classified as Wuhan, other cities in Hubei province, and other provinces. The second section—the KAP section—was further divided into the following three parts.

1. The knowledge part consisted of fourteen questions that attempted to test the COVID-19 knowledge of the survey participants. These questions were designed based on the second edition of the Health Education Manual that was published by the National Institute of Health Education of the National Health Commission in China; there were nine questions on epidemiological knowledge of COVID-19, and five regarding the prevention of COVID-19 virus infection. The knowledge questions were represented in a statement form, i.e., “COVID-19 can spread through person-to-person transmission,” and the participants were asked to choose right or wrong on these questions. An additional “do not know” option was also provided. One point was assigned to a correct response, and 0 points was assigned to “do not know” or wrong responses. The total score thus ranged from 0 to 14.
2. The attitude part had nine questions that assessed the attitudes of industry practitioners toward COVID-19; four questions were about their level of confidence regarding the successful control of the pandemic, and five were about their level of satisfaction toward the control measures taken by the government and their companies. The participants were asked to reflect their attitude on a five-point Likert scale, in which 1 represented “very low” and 5 represented “very high.”
3. The practice part contained eight questions on preventive measures, for instance, whether they wore an appropriate mask during their work. The participants were asked to report the behaviors taken by themselves to prevent infections on a five-point Likert scale, in which 1 represented “never do that” and 5 represented “always do that.”

All these KAP questions were reviewed by subject matter experts to enhance the adequacy and appropriateness, and a redundancy question was designed in the attitude part to help reduce social desirability bias in the responses.

### Data Collection and Analysis

A total of 785 responses were collected initially. After removing the incomplete and invalid (i.e., providing a different answer for redundancy question) ones, 702 responses were finally obtained. The data collection conformed to the ethics guidelines of Huazhong University of Science and Technology, and the confidentiality of the participants' data was protected by hiding the identity of the respondents. Then, statistical analysis was performed by using SPSS v.22.0. The normality of data was assessed by Shapiro-Wilk tests. Additionally, Pearson Chi-Square and Kruskal-Wallis tests were adopted to assess whether the KAP levels were statistically different across different demographic characteristics of respondents.

## Results

### Demographic Information of the Respondents

Of the 702 respondents, 588 (83.8%) were males and 114 (16.2%) were females; 94 were in Wuhan, 26 were in other cities in Hubei province, and the remaining 582 were in other provinces. Other demographic characteristics of the samples are summarized in Table 1.

**Table 1:** Characteristics of survey participants (N = 702).

**Characteristics**		**Number**	**Percentage (%)**
Gender	Male	588	83.76%
	Female	114	16.24%
Age	<25	78	11.11%
	25–30	274	39.03%
	31–40	273	38.89%
	41–50	65	9.26%
	>50	12	1.71%
Years of work experience	≤5	259	36.89%
	6–10	243	34.62%
	11–15	100	14.24%
	16–20	49	6.98%
	≥21	51	7.27%
Stakeholder	Developer	49	6.98%
	Designer	103	14.67%
	Main contractor	504	71.80%
	Sub-contractor	30	4.27%
	Others	16	2.28%
Location of the project	Wuhan	94	13.39%
	Other cities in Hubei Province	26	3.70%
	Other provinces	582	82.91%
Type of the project	Residential	294	41.88%
	Commercial	185	26.35%
	Industrial	45	6.41%
	Infrastructure	167	23.79%
	Others	11	1.57%

### Knowledge of the Respondents

**Figure 1:**
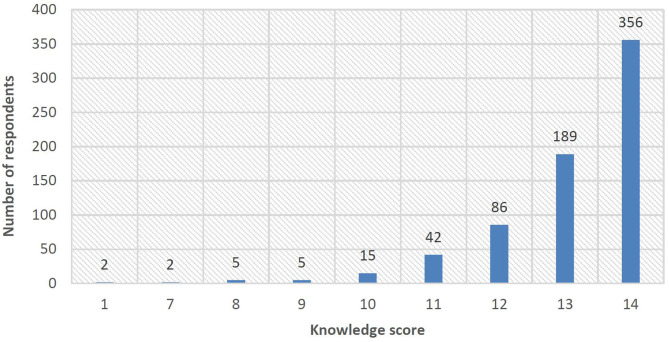
Distribution of knowledge scores.

Table 2 and Figure 1 depict the knowledge of respondents toward COVID-19. The average knowledge score was 13.09 (SD = 1.36), and eight questions had an accuracy rate of over 95%. Such results indicated good knowledge of industry practitioners toward COVID-19. However, two knowledge questions deserved attention, i.e., “Humans are universally susceptible to COVID-19” and “Asymptomatic infection is contagious,” since over 15% of the respondents reported wrong or unsure answers.

**Table 2:** Knowledge of respondents toward COVID-19.

**Questions**	**Yes (%)**	**No (%)**	**Don't know (%)**
Humans are universally susceptible to COVID-19.	595 (84.76%)	71 (10.11%)	36 (5.13%)
COVID-19 can spread through person-to-person transmission.	694 (98.86%)	5 (0.71%)	3 (0.43%)
The general observation period of COVID-19 is 14 days.	661 (94.16%)	15 (2.14%)	26 (3.70%)
The transmission modes of COVID-19 include droplet transmission, contact transmission, and aerosol transmission.	681 (97.01%)	12 (1.71%)	9 (1.28%)
Not all people with COVID-2019 will develop to severe cases.	680 (96.87%)	12 (1.71%)	10 (1.42%)
Asymptomatic infection is contagious.	596 (84.90%)	33 (4.70%)	73 (10.40%)
Cured patients are still at risk of reinfection.	604 (86.04%)	35 (4.99%)	63 (8.97%)
The main clinical symptoms of COVID-19 are fever, fatigue, and dry cough.	696 (99.15%)	1 (0.14%)	5 (0.71%)
Until June 2020, there is no effective cure for COVID-19.	668 (95.16%)	8 (1.14%)	26 (3.70%)
People can wear medical masks to prevent infection.	671 (95.58%)	17 (2.42%)	14 (1.99%)
Masks should be replaced after contamination or moisture.	689 (98.15%)	6 (0.85%)	7 (1.00%)
Used masks should be discarded as hazardous waste.	678 (96.58%)	19 (2.71%)	5 (0.71%)
75% alcohol and chlorine-containing disinfectants can effectively eliminate the virus.	631 (89.89%)	33 (4.70%)	38 (5.41%)
Vinegar cannot effectively eliminate the virus.	642 (91.45%)	22 (3.13%)	38 (5.41%)

Differences in the knowledge scores across various demographic characteristics were evaluated by using Pearson Chi-Square and Kruskal-Wallis tests. As shown in Table 3, the knowledge scores were significantly different across ages, stakeholders, and project locations. However, no significant differences in the knowledge scores across genders, years of work experience, and project types were identified.

**Table 3:** Knowledge score of COVID-19 by demographic characteristics.^#^

**Characteristics**		**Mean (SD)**	***X*^**2**^**	***P***
Gender	Male	13.08 (1.41)	7.902	0.443
	Female	13.10 (1.11)		
Age	<25	12.90 (1.28)	10.472	0.033^*^
	25–30	13.26 (1.02)		
	31–40	13.08 (1.56)		
	41–50	12.72 (1.64)		
	>50	12.42 (1.93)		
Years of work experience	≤5	13.14 (1.09)	5.427	0.246
	6–10	13.16 (1.38)		
	11–15	13.01 (1.80)		
	16–20	13.06 (1.11)		
	≥21	12.63 (1.73)		
Stakeholder	Developer	13.02 (1.09)	11.258	0.024^*^
	Designer	12.92 (1.20)		
	Main contractor	13.14 (1.42)		
	Sub-contractor	12.90 (1.16)		
	Others	13.00 (1.55)		
Location of the project	Wuhan	12.81 (1.20)	13.437	0.001^*^
	Other cities in Hubei Province	12.96 (1.18)		
	Other provinces	13.14 (1.39)		
Type of the project	Residential	13.05 (1.44)	1.606	0.808
	Commercial	13.11 (1.25)		
	Industrial	13.02 (1.10)		
	Infrastructure	13.13 (1.45)		
	Others	13.27 (0.79)		

# *X^2^ for gender shows Pearson Chi-Square value and for other demographic characteristics shows Kruskal-Wallis H value*.

* *P ≤ 0.05 indicates significance*.

### Attitudes of the Respondents

**Figure 2:**
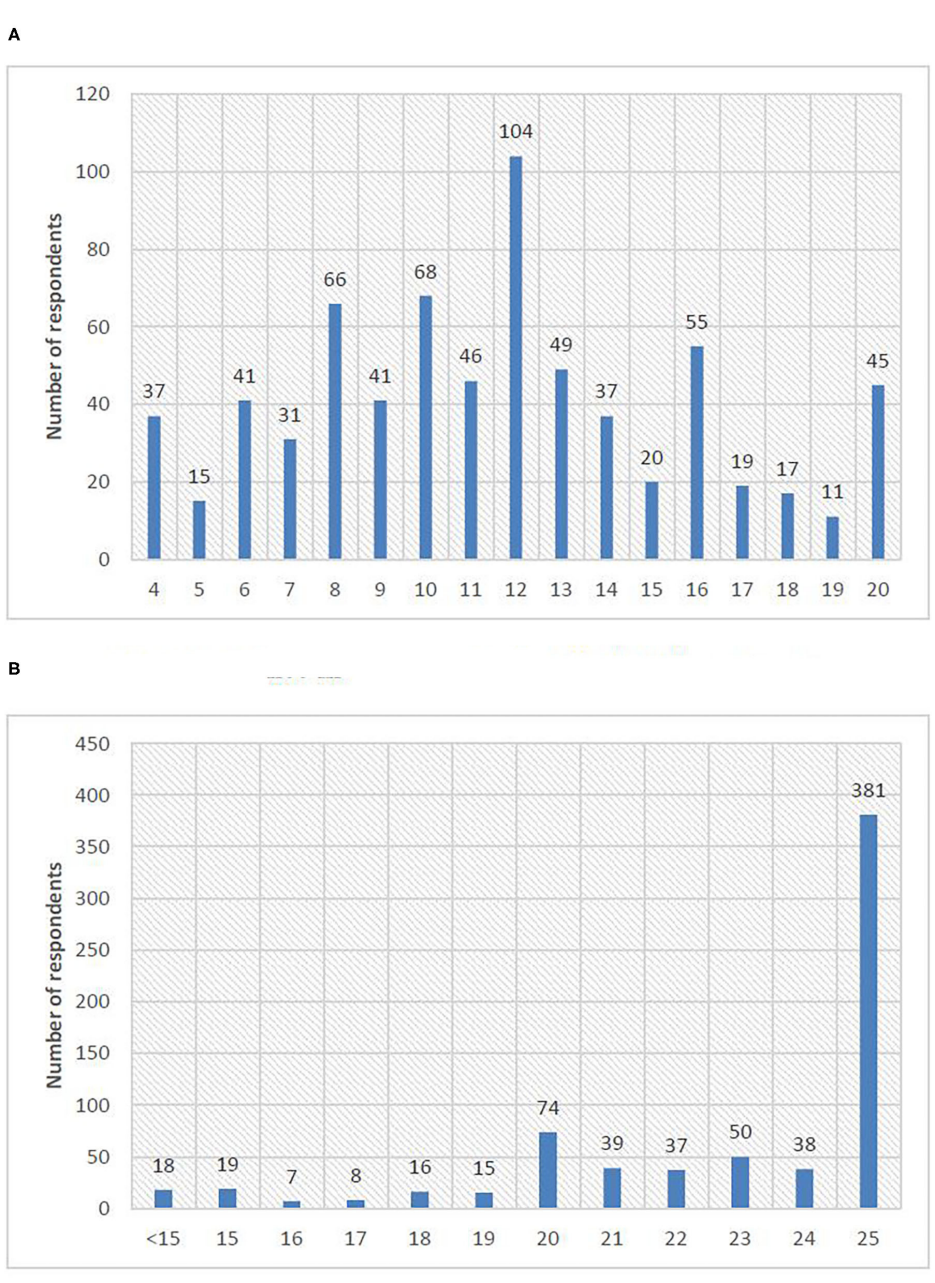
Distribution of attitude scores. **(A)** Confidence in overcoming the COVID-19 pandemic. **(B)** Satisfaction with the control measures.

Table 4 and Figure 2 reveal the respondents' attitudes toward COVID-19. The average scores on confidence in overcoming the COVID-19 pandemic and satisfaction with the control measures were 11.52 (SD = 4.26) and 22.70 (SD = 3.68), respectively, indicating an overall positive attitude that the pandemic would be successfully addressed. It is also encouraging to see the majority of the surveyed industry practitioners can effectively continue their work during the COVID-19 outbreak, and 619 out of all respondents had high-level satisfaction with the measures taken by the government and their companies in controlling the virus spread.

**Table 4:** Attitude of respondents toward COVID-19.

**Questions**		**1 (%)**	**2 (%)**	**3 (%)**	**4 (%)**	**5 (%)**
Confidence in overcoming the COVID-19 pandemic	I will not be infected with COVID-19.	160 (22.79%)	116 (16.52%)	242 (34.47%)	81 (11.54%)	103 (14.67%)
	My colleagues will not be infected with COVID-19.	168 (23.93%)	143 (20.37%)	228 (32.48%)	77 (10.97%)	86 (12.25%)
	I have no worry of going to work during the COVID-19 outbreak.	127 (18.09%)	145 (20.66%)	239 (34.05%)	103 (14.67%)	88 (12.54%)
	I do not feel tired at work during the COVID-19 outbreak.	74 (10.54%)	107 (15.24)	243 (34.62%)	136 (19.37%)	142 (20.23%)
Satisfaction with the control measures	I am satisfied with my company's requirements for wearing masks and temperature measurement.	15 (2.14%)	1 (0.14%)	20 (2.85%)	96 (13.68%)	570 (81.20%)
	I am satisfied with my company's regular disinfection.	15 (2.14%)	6 (0.85%)	44 (6.27%)	142 (20.23%)	495 (70.51%)
	I am satisfied with my company's preparation of anti-epidemic resources.	24 (3.42%)	12 (1.71%)	53 (7.55%)	150 (21.37%)	463 (65.95%)
	I think the government has timely publicized relevant information on COVID-19.	14 (1.99%)	11 (1.57%)	51 (7.26%)	168 (23.93%)	458 (65.24%)
	I think the control measures taken by the government are effective.	17 (2.42%)	7 (1.00%)	54 (7.69%)	163 (23.22%)	461 (65.67%)

Significant differences in the respondents' attitudes across their demographic characteristics were identified. As shown in Table 5, the attitude significantly differed across years of work experience. In addition, the respondents who worked in Wuhan had significantly lower satisfaction with the control measures than that of respondents in other cities. The satisfaction with the control measures also varied significantly across stakeholders.

**Table 5:** Attitude score by demographic characteristics.

**Characteristics**		**Confidence in overcoming the COVID-19 pandemic**		**Satisfaction with the control measures**	
		**Mean (SD)**	***X*^**2**^ (P)**	**Mean (SD)**	***X*^**2**^ (P)**
Gender	Male	11.563 (4.320)	0.178 (0.673)	22.759 (3.621)	0.934 (0.334)
	Female	11.325 (3.925)		22.404 (4.002)	
Age	<25	11.756 (4.316)	10.880 (0.028)^*^	22.603 (4.069)	3.556 (0.469)
	25–30	10.974 (4.259)		22.693 (3.428)	
	31–40	11.733 (4.232)		22.725 (3.770)	
	41–50	12.462 (4.280)		23.092 (3.390)	
	>50	12.750 (2.989)		20.833 (5.875)	
Years of work experience	≤5	11.100 (4.168)	10.378 (0.035)^*^	22.827 (3.219)	10.735 (0.030)^*^
	6–10	11.362 (4.270)		22.193 (4.302)	
	11–15	12.140 (4.459)		23.550 (2.904)	
	16–20	12.490 (4.416)		23.163 (2.889)	
	≥21	12.314 (3.834)		22.373 (4.418)	
Stakeholder	Developer	10.531 (3.836)	3.386 (0.495)	22.796 (3.014)	11.323 (0.023)^*^
	Designer	11.272 (4.282)		21.971 (3.932)	
	Main contractor	11.637 (4.293)		22.885 (3.643)	
	Sub-contractor	11.933 (3.973)		22.367 (3.378)	
	Others	11.875 (4.689)		21.938 (5.234)	
Location of the project	Wuhan	11.894 (3.650)	1.373 (0.503)	21.926 (3.699)	11.279 (0.004)^*^
	Other cities in Hubei Province	11.192 (3.720)		22.154 (4.370)	
	Other provinces	11.479 (4.371)		22.851 (3.638)	
Type of the project	Residential	11.320 (4.338)	1.960 (0.743)	22.616 (3.969)	0.958 (0.916)
	Commercial	11.838 (4.358)		22.514 (3.887)	
	Industrial	11.356 (3.199)		22.644 (3.791)	
	Infrastructure	11.587 (4.200)		23.012 (2.912)	
	Others	11.455 (5.298)		23.636 (2.157)	

* *P ≤ 0.05 indicates significance*.

### Practices of the Respondents

The correlation coefficient between knowledge and practices is 0.91 (*P* = 0.016), revealing the significant association between them, i.e., those who have a better knowledge of COVID-19 have taken more preventive measures. Overall, 93.73% of the survey participants always attended the health education sessions organized by their companies. 89.03% of the respondents reported that they kept their work environment clean and ventilated; 74.79% washed their hands frequently during work hours. However, there were still some practices that were not widely adopted by industry practitioners. 32.91% of the respondents reflected that they did not conduct a frequent self-check of their body temperature, and 32.48% did not research the latest information about COVID-19. In addition, 25.21 and 16.52% of the respondents did not wear a face mask and keep sufficient social distance respectively, which potentially increased the chance of being exposed to the virus. Moreover, Table 6 shows that females maintained safety practices better than males, and the respondents in Wuhan performed better virus preventive practices than those in other cities in China.

**Table 6:** Practice score by demographic characteristics.^#^

**Characteristics**		**Mean (SD)**	***X*^**2**^**	***P***
Gender	Male	34.357 (5.093)	37.614	0.050^*^
	Female	35.289 (4.367)		
Age	<25	34.808 (3.871)	5.348	0.253
	25–30	34.128 (4.928)		
	31–40	34.740 (5.352)		
	41–50	34.692 (4.740)		
	>50	35.000 (5.924)		
Years of work experience	≤5	34.073 (4.633)	9.773	0.044^*^
	6–10	35.021 (4.717)		
	11–15	34.190 (6.345)		
	16–20	34.429 (4.770)		
	≥21	34.980 (5.159)		
Stakeholder	Developer	33.714 (5.955)	2.506	0.644
	Designer	34.874 (4.614)		
	Main contractor	34.538 (5.039)		
	Sub-contractor	34.033 (4.106)		
	Others	34.563 (4.305)		
Location of the project	Wuhan	35.926 (4.615)	13.808	0.001^*^
	Other cities in Hubei Province	35.385 (3.900)		
	Other provinces	34.241 (5.056)		
Type of the project	Residential	34.374 (4.971)	1.707	0.789
	Commercial	34.492 (4.970)		
	Industrial	34.889 (5.314)		
	Infrastructure	34.611 (5.014)		
	Others	35.273 (4.941)		

# *X^2^ for gender shows Pearson Chi-Square value and for other demographic characteristics shows Kruskal-Wallis H value*.

* *P ≤ 0.05 indicates significance*.

## Discussion

The emergence of COVID-19 continues to threaten public health in the global community. In response to this crisis, the construction industry has had a vital role in building hospitals and essential infrastructure that helped society to recover from the pandemic. However, the industry itself has been more seriously affected than many other economic sectors. Direct impacts caused by the COVID-19 crisis ranged from a slowdown of resource supply to terminations of entire projects. A recent report published by McKinsey & Company suggested that a fast return to pre-pandemic levels seems unlikely for the construction industry, and the industry must adapt to a “next normal” (14). This situation became much more severe in China since China has the world's largest construction market. Facing the sustained business downturn, the industry needs to assess its preparedness and arrange proper prevention and control measures, which call for the collation of industry practitioner's KAP toward COVID-19.

To the best of our knowledge, the present study is the first to investigate the KAP toward COVID-19 among construction industry practitioners in China. The differences in knowledge, attitudes, and practices were observed across various demographic characteristics, and the gaps were also identified so that essential health precautions can be enhanced to protect the practitioners from infection.

A high correct rate of COVID-19 knowledge was unsurprising because the survey was conducted in the middle stage of the COVID-19 outbreak. From December 2019 to June 2020, the government continuously provided the most up-to-date information of COVID-19 to the public through several social media channels. Nevertheless, respondents still showed a lack of understanding of who is susceptible to COVID-19 and whether asymptomatic infection is contagious. Such important knowledge gaps were also reported in Al-Hanawi et al. (15), Hayat et al. (16), and many other studies that targeted different groups of people in other parts of the world. Considering the world is still threatened by the COVID-19 pandemic, knowledge transmission strategies should be further explored to consolidate the knowledge of the public. On the other hand, since over 65% of the survey participants said they would search for information related to COVID-19, it is necessary to reduce the widespread levels of misinformation (17).

In a survey conducted by Zhong et al. (9), 90.8% of the surveyed Chinese residents believed that COVID-19 would be successfully controlled. Yue et al. (18) also reported that Chinese urban and rural residents had a positive attitude toward the pandemic. Such optimistic attitudes were in agreement with our findings. Additionally, the high-level satisfaction with government efforts can be attributed to the fact that the Chinese government has taken an active role in fighting against the COVID-19 pandemic. Several measures, such as imposing a strict lockdown in Wuhan and the development of Fangcang Hospital, have been considered effective in helping to control the virus spread as much as possible (19, 20). China has made strategic achievements in overcoming the COVID-19 disruption and has gradually resumed social and economic activities. Nevertheless, this study found that the construction industry practitioners in Wuhan have a lower satisfaction level than those in other cities. More investigations need to be conducted before reaching an in-depth understanding of this issue.

The majority of the surveyed industry practitioners took different preventive measures to prevent possible infection, and it is especially delighting to see that a large proportion of the respondents actively attended the health education training that has been acknowledged as a common but important activity for COVID-19 prevention (21). Overall, our findings on respondents' practices align with the findings of Li et al. (22) who reported better virus prevention practices of respondents living in Hubei province than those living in other provinces. However, it is notable that a handful of survey participants omitted the importance of checking body temperature and wearing face masks. The shortage of qualified resources (such as face masks and thermometers) during the COVID-19 outbreak could be one of the main reasons for such gaps in practices. Another serious issue is that over 16% of the respondents failed in maintaining safe social distance. Previous studies conducted in different countries found that keeping social distance was among the main preventive measures for the general population (23, 24). The unique characteristics of construction project delivery require that the practitioners not only work in independent offices but have to collaborate with each other on the construction site. Therefore, it is difficult for them to maintain a safe social distance and avoid face-to-face contact throughout the project. All these exposed practice gaps have implications for both enhanced short-term and long-term control measures in the construction industry. Where work continues, health and safety risk assessments need to be frequently conducted in order to block the virus transmission route and provide a safe working environment for employees.

This study enriched the data on the KAP of a specific group of people, i.e., construction industry practitioners in China, toward COVID-19. However, this study was prone to two limitations. First, due to the limited sample representativeness, caution should be taken when generalizing the findings to the construction industries in other regions. Second, it was unavoidable that some respondents would give socially desirable responses that did not reflect the actual situation. This limitation can be reduced by triangulation with on-site observation, and other data collection methods. However, the authors found it was difficult to conduct site visits since many companies showed reluctance to let people outside their projects enter the jobsite.

## Conclusions

This study contributes to the global research effort in helping the construction industry to fight against the COVID-19 pandemic. It enriched the understanding of the KAP of construction industry practitioners toward COVID-19 through a comprehensive survey investigation. The findings indicated that most of the respondents have a good level of knowledge and are generally positive about the eradication of the pandemic. The respondents have taken precautious roles to protect themselves from infection. The identification of current KAP status also highlighted the gaps in respondents' knowledge and practices, which should be addressed to reduce COVID-19 spread in the construction industry.

Since the construction industry is vulnerable to the COVID-19 crisis, further studies could be conducted to assess the impacts of COVID-19 on the productivity of the construction industry and explore strategies to help the industry to deal with the disruptions.

## Data Availability Statement

The raw data supporting the conclusions of this article will be made available from the corresponding author by request.

## Ethics Statement

The studies involving human participants were reviewed and approved by the approval for this research was given by the Huazhong University of Science and Technology, China. Written informed consent for participation was not required for this study in accordance with the national legislation and the institutional requirements.

## Author Contributions

KC and LM: conceptualization and resources. KC: methodology, writing—review and editing, and project administration. LZ: formal analysis, investigation, writing—original draft preparation, and visualization. All authors have read and agreed to the published version of the manuscript.

## Conflict of Interest

The authors declare that the research was conducted in the absence of any commercial or financial relationships that could be construed as a potential conflict of interest.
